# Performance evaluation of the highly sensitive histidine‐rich protein 2 rapid test for *plasmodium falciparum* malaria in North-West Tanzania

**DOI:** 10.1186/s12936-020-03568-z

**Published:** 2021-01-22

**Authors:** Alphaxard Manjurano, Justin J. Omolo, Eric Lyimo, Donald Miyaye, Coleman Kishamawe, Lucas E. Matemba, Julius J. Massaga, John Changalucha, Paul E. Kazyoba

**Affiliations:** 1 Mwanza Medical Research Center, National Institute for Medical Research, Mwanza, Tanzania; 2 National Institute of Medical Research, Head Quarters, P.O. Box 9653, Dar es Salaam, Tanzania; 3 Dodoma Medical Research Center, National Institute for Medical Research, Dodoma, Tanzania

**Keywords:** HSPf, SD Bioline Pf, Microscopy, Sensitivity, Specificity, *Plasmodium falciparum*

## Abstract

**Background:**

Precise detection of *Plasmodium* infections in community surveys is essential for effective malaria control. Microscopy and rapid diagnostic tests (RDTs) are the major techniques used to identify malaria infections in the field-based surveys. Although microscopy is still considered as the gold standard, RDTs are increasingly becoming versatile due to their rapid and adequate performance characteristics.

**Methods:**

A malaria prevalence cross-sectional survey was carried out in north-western Tanzania in 2016, aimed at appraising the performance of high sensitivity *Plasmodium falciparum* (HSPf) tests compared to SD Bioline Pf and microscopy in detecting *P. falciparum* infections. A total of 397 individuals aged five years and above were tested for *P. falciparum* infections. The sensitivity, specificity, positive, and negative predictive values (PPV and NPV) of microscopy, Pf RDT and HSPf RDT was determined using PCR as the gold standard method.

**Results:**

The prevalence of *P. falciparum* infections determined by microscopy, SD Bioline Pf, HSPf and PCR was 21.9, 27.7, 33.3 and 43.2%, respectively. The new HSPf RDT had significantly higher sensitivity (98.2%) and specificity (91.6%) compared to the routinely used SD Bioline Pf RDT(P < 0.001). The positive predictive value (PPV) was 81.8% and the negative predictive value (NPV) was 99.2% for the routinely used SD Bioline Pf RDT. Moreover, HSPf RDT had sensitivity of 69% and specificity of 76.8% compared to microscopy. The PPV was 45.5% and the NPV was 89.8% for microscopy. Furthermore, the analytical sensitivity test indicated that the newly developed HSPf RDT had lower detection limits compared to routinely used SD Bioline RDT.

**Conclusions:**

HSPf RDT had better performance when compared to both microscopy and the currently used malaria RDTs. The false negativity could be associated with the low parasite density of the samples. False positivity may be related to the limitations of the expertise of microscopists or persistent antigenicity from previous infections in the case of RDTs. Nevertheless, HS PfRDT performed better compared to routinely used Pf RDT, and microscopy in detecting malaria infections. Therefore, HS Pf RDT presents the best alternative to the existing commercial/regularly available RDTs due to its sensitivity and specificity, and reliability in diagnosing malaria infections.

## Background

Recent reports have showed a significant decline of malaria burden in some endemic countries, whereby the incidences have declined by over 50% in the past two decades [1]. However, the 2018 World Malaria Report of the World Health Organization (WHO), indicates that malaria remains one of the major public health problems in many countries. Malaria is responsible for more than 405,000 deaths worldwide [2], with 93% of these reported from sub-Saharan Africa [3]. The report has further indicated that, morbidity due to malaria was about 228 million cases annually, with the majority of these occurring in non-immune children under 5 years old and pregnant women [1].

 Various efforts have been made to reduce the burden of malaria in the world, particularly in sub-Saharan Africa. The last decade has seen a substantial progress made through adoption and scale-up of various malaria interventions [4–8]. Despite these gains, challenges such as emergence and spread of malaria parasites resistance to drugs [9–11] as well as mosquito resistance to insecticides threatens to reverse the progress made [12, 13]. Additionally, the cost of maintaining existing control efforts and extending high level control to high burden countries indefinitely will be challenging. To prevent resurgence and hasten progress, the Bill and Melinda Gates Foundation has developed a Strategy for Malaria Eradication termed, “Accelerate to Zero”. Accelerate to Zero is founded on the principle that malaria eradication requires elimination of parasites from the human population [14]. To achieve this, passive case detection of symptomatic individuals must be augmented by testing and treatment strategies that target the asymptomatic transmission reservoir [15–17]. Available commercial rapid diagnostic tests (RDTs) lack sufficient limit of detection (LOD) to identify all individuals with transmissible infections [17–20]. Thus, more sensitive diagnostic tests with improved LOD are needed to correctly identify infected individuals with low parasite densities who contribute to transmission [14, 20]. Microscopic examination of Giemsa-stained blood smears remains the gold standard for malaria diagnosis despite its technical challenges and demand for trained personnel. However, malaria rapid diagnostic tests (RDT) have become an important tool in interventions against malaria, where nearly half a billion cassettes were sold globally in 2018 [2]. Recent inventions have made possible for the RDT to be highly sensitive and effective in diagnosing latent and active *Plasmodium* infections [21, 22]. The RDTs were first developed in early 1990 s, and were mostly based on the detection of *Plasmodium* antigens, the *Plasmodium* lactate dehydrogenase (pLDH) and *P. falciparum* histidine-rich protein 2(HRP2) [23]. The HRP2-based RDTs are most common, and a number of manufactured kits have already been evaluated. These include: Paracheck Pf RDT (Orchid Biomedical Systems, Goa, India) and SD Bioline Malaria Ag Pf RDT (SD Bioline Korea), Determine ^TM^ Malaria Pf test (Abbott Laboratories, Tokyo, Japan), and ParaSight-F test3 (Becton Dickinson, Sparks Maryland 21,152 USA) [24–26]. Besides the HRP2 *P. falciparum* based rapid test, there are RDT kits capable of detecting more than one *Plasmodium* antigen and species, such the combo test. However, ongoing field evaluation have reported poor limit of detection for most rapid tests. Recently, manufacturers have developed highly sensitive RDTs based on HRP2 antigen detection, including the Alere™ Malaria Ag Pf Ultra-Sensitive rapid diagnostic test (SD/Alere, Korea) and SD Bioline Malaria Ag Pf High Sensitive. These second generation PfHRP2-based RDT kits have been reported to have a ten-fold sensitivity over their predecessors and make them highly suitable for malaria control and elimination programmes [21, 22, 24], but these tests have not undergone field evaluation especially in the areas where malaria is endemic.

The limitations shown by current rapid tests for malaria detection, has necessitated further innovations on sensitivity and reliability of the test kits [27]. Increasing sensitivity of the malaria rapid test kits not only will reduce the waiting time, but will improve on the reliability of results, and reduce the proportion of false negatives. This study was aimed at determining the performance characteristics (sensitivity, specificity and analytical sensitivity) of the new RDT SD Bioline Malaria Ag Pf High Sensitive (hereafter ‘HSPf’), when compared with the current commercially available, routinely used Pf RDT and blood smear microscopic examination using blood specimens collected during a survey.

### Methods

#### Study design

This was a cross-sectional study, which was conducted for two months. The study participants were patients aged ≥ 5 years presenting with clinical features suggestive of malaria at the dispensaries, and the outpatient departments at the health centres and district hospitals.

### Study area

The study was conducted in three sites in Tanzania, namely Magu District Council in Mwanza region, and Geita Town Council and Geita District Council in Geita region. Selection of the two regions was based on the Tanzania HIV and Malaria Indicator Survey of 2011/2012 [3] which showed Geita had a malaria prevalence of 32% in under 5 years, while Mwanza had 19% [3]. Furthermore, the regions are considered as moderate (Mwanza) to high malaria transmission (Geita) areas, with stable transmission intensity. The main peak of malaria transmission occurs at the end of the rainy season, between March and May. A study from the same geographical area reported that 97% of malaria infections were due to *P. falciparum*, but *Plasmodium malariae* and *Plasmodium ovale* are present either alone or in mixed infections (*Plasmodium vivax* is uncommon) [28].

### Sample size

Taking the prevalence of malaria of 29% in 2015, the desired probability of 5% and the level of confidence of the selected sample size of 95%. As suggested by the manufacturer (Standard Diagnostics Bioline), a minimum of 300 clinical samples was obtained from subjects presenting with symptoms relating to malaria, as described in the inclusion and exclusion criteria. It was thus estimated that, from each of the three study sites a minimum of 100 specimens would be collected for testing the sensitivity of the HSPf.

### Inclusion and exclusion criteria

#### Inclusion criteria

Since this study involved patients with symptoms related to malaria, the following criteria guided the selection of patients;

1. Patients aged 5 years and above were recruited for the study.
2. History of fever or other symptoms suggestive of malaria during the past 24 hours with or without fever at presentation (axillary ≥ 37.5 °C).
3. Informed consent from the participants/parent or guardian of children.

#### Exclusion criteria

1. Patients who have taken malaria drugs within 4 weeks before commencement of the study.
2. Patients below 5 years of age.
3. Presence of febrile conditions due to diseases other than malaria (e.g. measles, acute lower respiratory tract infection, severe diarrhoea with dehydration) or other known underlying chronic or severe diseases (e.g. cardiac, renal and hepatic diseases, HIV/AIDS).
4. Presence of general danger signs and hospitalization with multiple blood sampling.

### Microscopy

Thick and thin blood smears were made on the same slide, air dried and transported to the NIMR Mwanza laboratory where they were stored. The slides were later stained with 10% Giemsa for 15 minutes and examined for malaria parasites by two independent technicians (double blind). The counts of the two technicians were accepted and used to calculate the average parasite density.

Asexual parasitaemia was quantified against 200 to 500 leucocytes, assuming a white blood cell count of 8,000/µl as recommended by the WHO [29]. A slide was considered negative if no parasite was seen when 500 leucocytes were counted. Quality control readings were performed in randomly selected samples.

The Pf RDT and HSPf RDT tests were performed according to the manufacturer’s instructions (Standard Diagnostics, Inc., Korea, www.standardia.com).

### **DNA extraction and PCR amplification for*Plasmodium*species**

Approximately 3 ml of venous blood was collected from participants into EDTA vacutainer tubes. Using a Pasteur pipette, blood was taken from the vacutainer tube and one drop was placed on each malaria RDT cassette and results were read after 30 minutes. Participants with a positive test result on the routinely used RDT were treated, in line with Tanzanian national treatment guidelines. DNA was extracted from remaining whole blood using the QIAamp DNA Mini Kit (QIAGEN, UK), following manufacturer’s instructions. A nested PCR was used to amplify species-specific sequences of the small sub-unit ribosomal ribonucleic acid (18S SSU rRNA) genes of *P. falciparum* as described [30, 31]. DNA from the culture of *P. falciparum* (3D7strain), DNA from blood samples of an individual never exposed to malaria, and PCR water were used as positive and negative controls and were included in each set of PCR for quality control. The PCR was carried out in a thermo-cycler (PTC-0240, The DNAengine® Thermal Cycler, Bio-Rad, Hercules, USA), followed by gel electrophoresis to determine whether blood sample was individuals as either parasite positive or negative.

### Analytical sensitivity

The analytical sensitivity test was conducted to determine and compare the detection limit of the HSPf RDT and the routinely used Pf RDT. Two replicates of blood specimens with higher, mid and low parasitaemia were used in the assay. The two-fold serial dilutions were done using a malaria negative whole blood specimen. Prior to their use in the assay, the malaria negative status of the diluents was confirmed using the HSPf RDT, the Pf RDT and blood smear slides.

### Statistical analysis

Data were analysed using STATA (StataCorp, Texas, USA) software version 12. Sensitivity, specificity, positive and negative predictive values (PPV and NPV) of microscopy, Pf RDT and HSPf RDT, were determined using PCR as the gold standard, using 2 × 2 contingency tables and compared using the McNemar’s test (sensitivity and specificity) for paired data [32]. Exact 95% confidence intervals (95 CI) were calculated for each measure listed above. For each of the two diagnostic techniques, the Pearson Chi-squared test was used to assess difference in sensitivity, specificity, and predictive values across the three age groups. Statistical significance was set at p < 0.05.

## Results

A total of 397 study participants aged 5 years and above were screened for malaria parasites. Among study participants, 57.2% (227/397) were females and the median age of participants was 24 years (IQR, 13–40). Participants from Magu district were younger than those from Geita district (Table 1).

**Table 1:** Distribution of study participants by sex and age

District	Male		Female		Age (years) Median (IQR)	Total
	n	%	n	%		
Magu	65	43.9	83	56.1	13 (9–36.5)	148
Geita TC	46	43	61	57	26 (20–40)	107
Geita DC	59	41.5	83	58.5	29 (20–42)	142
Total	170	42.8	227	57.2	24 (13–40)	397

Out of 397 blood samples tested for malaria, 110 (27.7%) and 132 (33.3%) were positive for *P. falciparum* infections by routinely used Pf RDT and HSPf RDT, respectively. While a total of 397 blood samples tested for malaria by PCR, 171 (43.2%) were positive for *P. falciparum* infections. Three blood samples were positive for *P. malariae* and one blood sample was positive for *P. ovale*. Moreover, 87 (21.9%) out of 397 blood samples were positive by microscopy (Table 2).

**Table 2:** Comparison of malaria parasite positivity by District and RDTs, microscopy and PCR techniques

District	Parasite prevalence				P-value
	SD Pf,%(n/N)	HS Pf,%(n/N)	Microscopy,% (n/N)	PCR,% (n/N)	
Magu	37.8 (56/148)	43.2 (64/148)	30.4 (45/148)	51.4 (76/148)	0.0002
Geita TC	20.6 (22/107)	29 (31/107)	20.6 (22/107)	42.1 (45/107)	0.0028
Geita DC	22.5 (32/142)	26.1 (37/142)	13.5 (20/142)	38.0 (54/142)	< 0.0001
Total	27.7 (110/397)	33.3 (132/397)	21.9 (87/397)	44.1 (175/397)	< 0.0001

Furthermore, 21 individuals (5.3%) and 8 individuals (2.1%) had *P. malariae* and *P. ovale* infections, respectively, detected by PCR. Of the 21 individuals who tested positive for *P. malariae*, 18 had mixed infection of *P. falciparum* + *P. malariae*, whereas 8 individuals who tested positive for *P. ovale* infections, 7 had mixed infection of *P. falciparum* + *P. ovale*. Moreover, three individuals had triple mixed infections of *P. falciparum*, *P. malariae* + *P. ovale*. The parasite density for *P. falciparum* ranged from 40–1,000,000 parasites/µL.

Parasite positivity was compared for the different testing methods. The HSPf RDT detected a higher proportion of parasite positivity than the two malaria tests routinely used, i.e. Pf RDT and microscopy. However, PCR detected the highest proportion of malaria parasite positivity than the other methods. PCR also detected more individuals with low parasitaemia (below 100 parasites/µL), but the difference was borderline (p = 0.090).

Microscopy correctly identified 69 out of 162 PCR-positive *P. falciparum* infections (42.6% sensitivity, 95% CI: 34.9–50.6) and 199 out of 217 PCR-negative samples (91.7% specificity, 95% CI: 87.2–95.0), with a PPV of 79.3% and NPV of 68.2%. The routinely used Pf RDT correctly identified 99 out of 171 PCR-positive *P. falciparum* infections (57.9% sensitivity, 95%CI: 50.1–65.4) and 214 out of 225 PCR-negative samples (95.1% specificity, 95% CI: 91.4–97.5), with a PPV of 90.0% and NPV of 74.8%. Whereas HSPf correctly identified 117 out of 171 PCR-positive *P. falciparum* infections (68.4% sensitivity, 95% CI: 60.9–75.3) and 210 out of 225 PCR-negative samples (93.3% specificity, 95%CI: 89.2–96.2), with a PPV of 86.6% and NPV of 79.5% (Table 3).

**Table 3:** Sensitivity, specificity, positive and negative predictive values of microscopy, Pf RDT and HSPf RDT compared to PCR as gold standard

	PCR Positive (n = 171)	PCR negative (n = 225)	Sensitivity % (CI95)	Specificity %(CI95)	PPV% (CI95)	NPV% (CI95)
Microscopy						
Positive (n = 87)	70	17	40.0 (32.6–47.7)	92.4 (88.0–95.5)	80.5 (70.6–88.2)	66.1 (60.6–71.4)
Negative (n = 310)	105	205				
Pf RDT						
Positive (n = 110)	99	11	56.5 (48.8–64.0)	95.0 (91.3–97.5)	90.0 (82.8–94.9)	73.5 (68.0–78.5)
Negative (n = 287)	76	211				
HSPf RDT						
Positive (n = 132)	117	15	69.9 (59.3–73.8)	93.2 (89.1–96.2)	88.6 (82–93.5)	78.1 (72.6–82.9)
Negative (n = 265)	58	207				

*PPV* positive predictive value,* NPV* negative predictive value,* CI95* 95% confidence interval

Comparing the sensitivity and specificity of HSPf RDT with standard Pf RDT, HSPf RDT had higher sensitivity (98.2%) and specificity (91.6%) compared to the routinely used Pf RDT (P < 0.001). The positive predictive value (PPV) was 81.8% and the negative predictive value (NPV) was 99.2% for the routinely used Pf RDT. Similarly, HSPf RDT had significantly higher sensitivity (69%) and specificity (76.8%) compared to microscopy. The PPV was 45.5% and the NPV was 89.8% for microscopy (P < 0.0001).

Although the sensitivity of the different tests decreased with increasing age of participants (> 15 years old), the sensitivity, specificity and predictive values of HSPf RDT was the highest among the three tests. The sensitivity of HSPf RDT among the different age groups was 80% (95% CI:64.4–90.9) in five to ten years old, 91.2% (95% CI: 76.3–98.1) in 11 to 15 years-old, and 55.7% (95% CI: 45.2–65.8) in those above 15 years of age (p < 0.0001). No significant change in the specificity of microscopy was observed across the three age groups. Similarly, the sensitivity of routinely used Pf RDT was lower in those above 15 years-old (42.3%, 95% CI: 32.3–52.7) than in five to 10 years (75,95% CI 58.8–87.3), and (82.4,95% CI 65.5–93.2) in those aged 11 to 15 years (p < 0.0001) (Table 4).

**Table 4:** Sensitivity, specificity and predictive values of microscopy, routine Pf RDT and HSPf RDT compared to PCR as gold standard by age group

Age	Sensitivity % (95CI)	Specificity % (95CI)	PPV % (95CI)	NPV % (95CI)
Microscopy				
5–10	52.5 (38.3–71.4)	93.7 (79.2–99.2)	91.3 (72.0–98.9)	61.2 (46.2–74.8)
11–15	58.3 (46.8–81.4)	100 (80.5–100)	100 (83.9–100)	51.6 (33.1–69.8)
> 15	28.2 (20.3–39.8)	91.4 (85.0–94.5)	65.1 (49.1–79.0)	69.1 (62.7–75.0)
P-value*	0.070	0.001	0.98	0.886
Pf				
5–10	75 (58.8–87.3)	93.8 (79.2–99.2)	93.8 (79.2–99.2)	75 (58.8–87.3)
11–15	77.8 (65.5–93.2)	75.0 (52.4–93.6)	87.5 (71.0–96.5)	60 (36.0–80.9)
> 15	41.4 (32.3–52.7)	97.1 (93.5–99.1)	89.1 (76.4–96.4)	75.2 (69.1–80.7)
P-value*	0.051	0.835	0.980	0.980
HSPf				
5–10	80.0 (64.4–90.9)	93.8 (79.2–99.2)	94.1 (80.3–99.3)	78.9 (62.7–90.4)
11–15	86.1 (76.3–98.1)	62.5 (41.0–86.7)	83.8 (68.0–93.8)	67 (38.4–88.2)
> 15	54.5 (45.2–65.8)	96.0 (91.9–98.4)	88.5 (77.8–95.3)	78.7 (72.6–84.1)
P-value*	0.196	0.640	0.945	0.999

Age shown in years,* PPV* positive predictive value,* NPV* negative predictive value, *95CI* 95% confidence interval

*Obtained from Pearson Chi-squared test

### Determination of minimum detection limit using specimens with higher parasitaemia

**Fig. 1:**
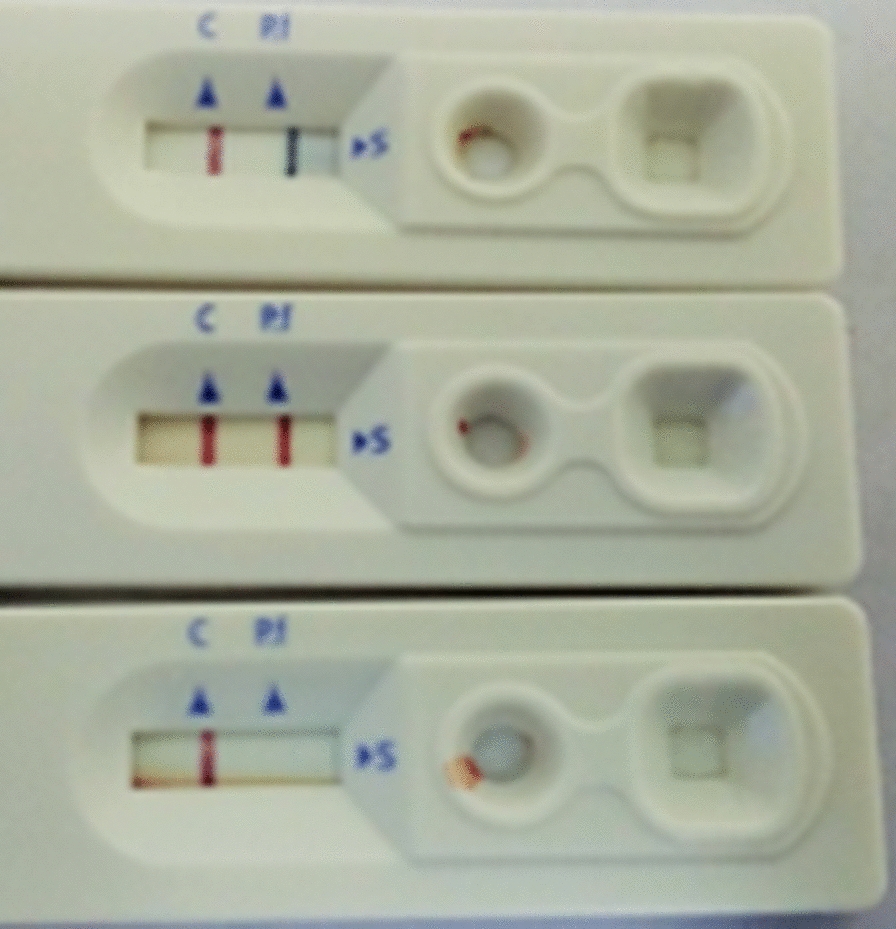
Confirmation of malaria positive specimen for the higher parasitaemia blood specimen using HS Pf RDT and SD RDT cassette. Showing determination of minimum detection limit using specimen with higher parasitaemia

**Fig. 2:**
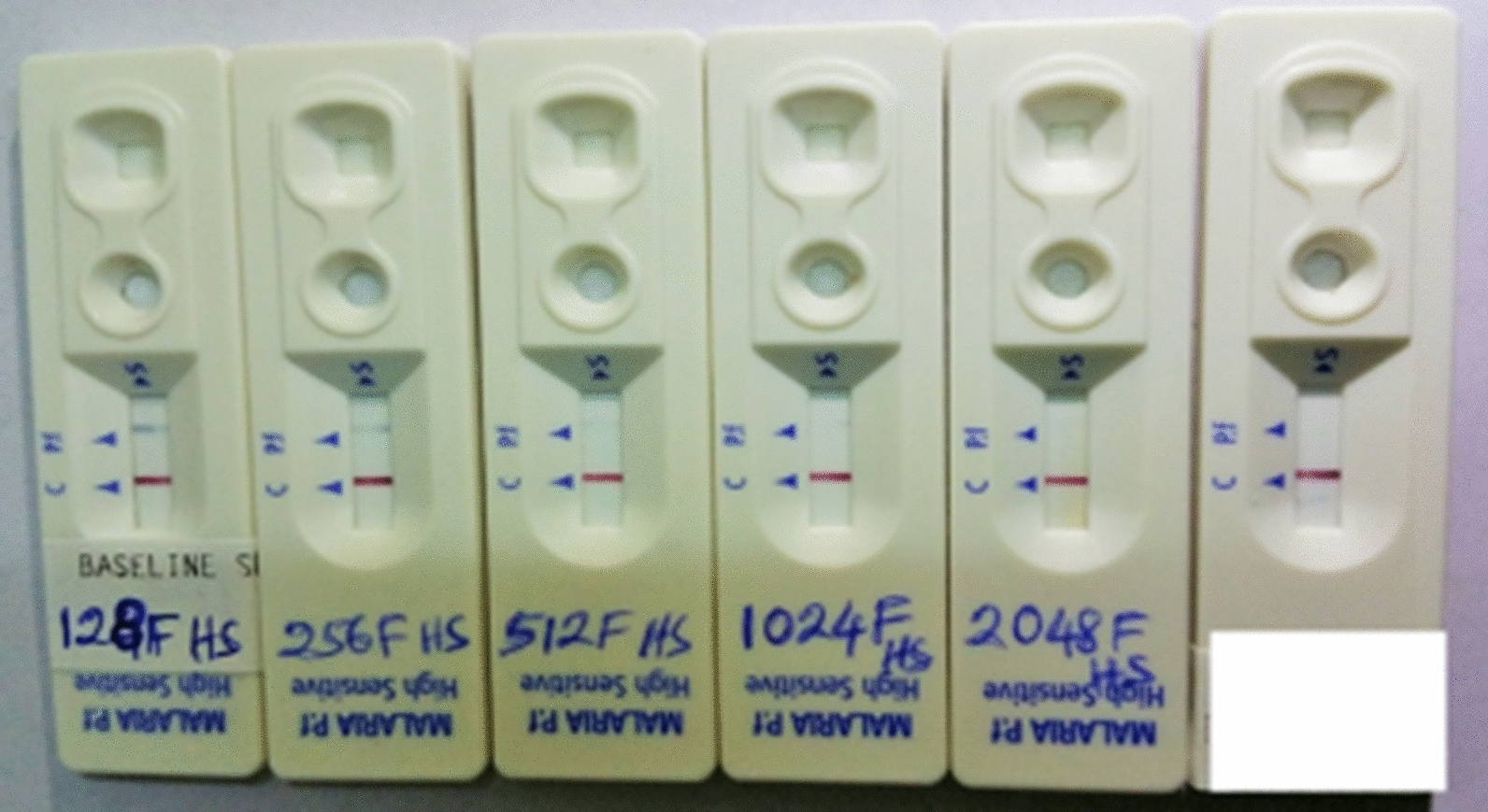
Analytical sensitivity test for both HSPf RDT and the currently used Pf RDT. A close range image of the last five HS RDT cassettes indicating the 1024F dilution detection limit, while the last cassette to the far right is reference test for negative reaction

**Fig. 3:**
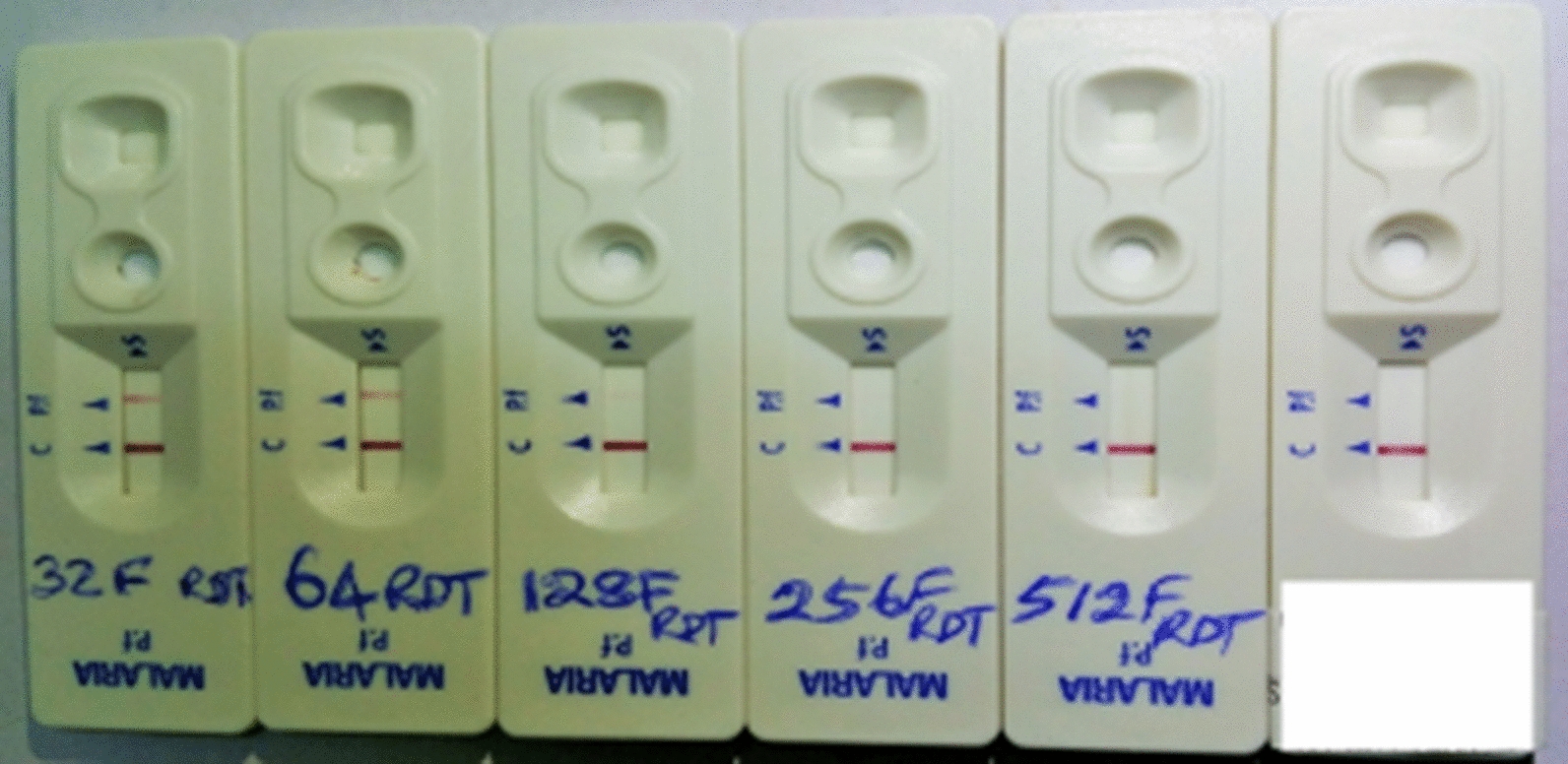
Analytical sensitivity test for both HSPf RDT and the currently used Pf RDT. A close range image of the last five Pf RDT cassettes indicating the 256F dilution as the minimum detection limit

The selected blood specimens with higher parasitaemia had parasite densities of between 874 parasites/200WBC and 1384 parasite/200WBC (Fig. 1). The analytical sensitivity test for both HSPf RDT and Pf RDT showed that the former had a minimum detection limit after 1024 two-fold dilutions, while Pf RDT had a limit of detection after 256 two-fold dilutions (Figs. 2 and 3).

### Determination of minimum detection limit using specimens with medium parasitaemia

The blood specimens with medium parasitaemia, between 468 and 574 parasites/200WBC, were used to study the sensitivity of the two RDTs. The HSPf RDT had a minimum detection limit after 64 two-fold dilutions, while that of the Pf RDT was 16 two-fold dilutions.

### Determination of minimum detection limit using specimens with low parasitaemia

The blood specimens with low parasitaemia, between 42 and 64 parasites/200WBC, were further used to study the sensitivity of two RDTs. The HSPf RDT had a minimum detection limit after 16 two-fold dilutions, while that of Pf RDT was 4 two-fold dilutions.

## Discussion

Detection of the malaria parasite reservoir is central for control given the renewed focus on transmission reduction leading to elimination. This study used venous blood sampling for determination of sensitivity and specificity of the different tests though both venous and DBS are used in the field setting. However, it should be noted that the decision of the choice of sampling methods depends on the choice of DNA extraction method which needs to be individualized depending on the level of laboratory facility, volume of testing, duration of samples storage and cost benefit analysis before it is adopted for use as it has shown in Uganda and Ethiopia [33, 34] and Equatorial Guinea [35]. Because this study aimed to compare sensitivity and specificity of several methods and using PCR as the gold standard, to other available methods of diagnosis of malaria; it should be noted that a very small amount of blood sample’s collected on filter paper and the possibility of asymptomatic *Plasmodium* infection, requires intense diagnosis to achieve accurate result with specificity and sensitivity. Therefore, it was necessary to use venous blood in order to avoid to perform several pricks on the same individual at the same time as it has been done in Thailand [36, 37], Brazil [38]and Tanzania [18].

In this study, the positivity by HSPf RDT was higher compared to microscopy and Pf RDT, though PCR had the highest positivity rates. However, compared to PCR as the gold standard, microscopy, Pf RDT and HSPf RDT detected only 40.9%, 57.9% and 68.4% of *P. falciparum* infections, respectively. In line with earlier studies, low parasite density might have affected the proportion of positive infections detected by microscopy and RDT [19]. Similar to observations made by Sousa-Figueiredo and colleagues, parasite density decreased with older age [39]. Accordingly, the present study found that sensitivity of microscopy and RDTs decreased with older age. This is also consistent with the fact that, in malaria endemic countries, acquired immunity in adult individuals is associated with the presence of submicroscopic infections that are more likely to be undetected by field microscopy or RDTs [18, 19, 40–42]. Moreover, the sensitivity is higher in children between 5 and 15 years because children will tend to have high parasite count as compared to adults and also immunity reaction is much more aggressive in children [43]. On the other hand, false negatives found by RDT may be explained by deletions or mutations within the *pfhrp-2* gene or by the prozone effect reported by others [44–47]. Nevertheless, RDTs were significantly more sensitive than microscopy, probably corroborating the capacity of RDTs to identify parasites below the threshold of microscopy as previously described [48, 49]. Furthermore, the false positives detected by microscopy may be explained by erroneous readings performed by the laboratory technicians, mistakenly counting dirt, cell debris and stain artefacts as malaria parasites. Moreover, false positive result by Pf RDT and HSPf RDT may be due to persistent antigenicity from previous infections and with cross-reactivity with autoantibodies, non-falciparum malaria and other infectious diseases [36, 40, 46, 49–53]. Given the low sensitivity and specificity of microscopy in this study, using it as gold standard for comparison would lead to the misclassification of samples and consequently misleading evaluation of the performance of RDTs. Although providing reliable epidemiologic information, the use of PCR is less likely to be implemented in studies conducted in developing countries due to the high costs involved [54–56].

The continued use of the current routinely used Pf RDT in detecting malaria in asymptomatic, low-density parasite infections poses a challenge in achieving malaria elimination. The strategy to eliminate malaria by 2030 will require highly sensitive diagnostic tools to detect the reservoir of low-density and submicroscopic parasite infections [57].

## Conclusions

Given the observed higher sensitivity values of HSPf RDT compared to Pf RDT and microscopy, the data presented here suggest that, the use of HSPf RDT to diagnose *P. falciparum* infections might improve detection over the current routinely used RDT or microscopy; but microscopy remains a preferable option, when parasite density needs to be determined in the absence of PCR. PCR allows for the detection of low-density infections and, even more importantly, mixed infections which are routinely missed in microscopy, making it an ideal confirmatory test for malaria diagnosis, but unfortunately difficult to implement on a large scale.

## Data Availability

The datasets used and/or analysed during the current study are available from the corresponding author on reasonable request.

## Abbreviations

DNA: Deoxyribose nucleic acid
EDTA: Ethyle diamine tetraacetic acid
HIV: Human Immunodeficiency Virus
AIDS: Acquired Immunodeficiency Syndrome
HS: High sensitive
LOD: Limit of detection
NPV: Negative Predictive Value
PCR: Polymerase Chain Reaction
Pf: *Plasmodium falciparum*
PfHRP2: *Plasmodium falciparum* HRP2
PPV: Positive Predictive Value
RDT: Rapid Diagnostic Test
WHO: World Health Organization
